# Population structure of elephant foot yams (*Amorphophallus paeoniifolius* (Dennst.) Nicolson) in Asia

**DOI:** 10.1371/journal.pone.0180000

**Published:** 2017-06-28

**Authors:** Edi Santosa, Chun Lan Lian, Nobuo Sugiyama, Raj Shekhar Misra, Patchareeya Boonkorkaew, Kanokwan Thanomchit

**Affiliations:** 1 Department of Agronomy and Horticulture, Faculty of Agriculture, Bogor Agricultural University, Bogor, West Java, Indonesia; 2 Asian Natural Environmental Science Center, The University of Tokyo, Nishitokyo-shi, Tokyo, Japan; 3 Department of Horticulture, Faculty of Agriculture, The University of Tokyo, Bunkyo-ku, Tokyo, Japan; 4 Central Tuber Crops Research Institute, Sreekariyam, Thiruvananthapuram, Kerala, India; 5 Department of Horticulture, Faculty of Agriculture, Kasetsart University, Bangkok, Thailand; National Cheng Kung University, TAIWAN

## Abstract

The corms and leaves of elephant foot yams (*Amorphophallus paeoniifolius* (Dennst.) Nicolson) are important foods in the local diet in many Asian regions. The crop has high productivity and wide agroecological adaptation and exhibits suitability for the agroforestry system. Although the plant is assumed to reproduce via panmixia, a comprehensive study on the genetic background across regions to enhance wider consumer palatability is still lacking. Here, ten informative microsatellites were analyzed in 29 populations across regions in India, Indonesia and Thailand to understand the genetic diversity, population structure and distribution to improve breeding and conservation programs. The genetic diversity was high among and within regions. Some populations exhibited excess heterozygosity and bottlenecking. Pairwise *F*_*ST*_ indicated very high genetic differentiation across regions (*F*_*ST*_ = 0.274), and the Asian population was unlikely to be panmictic. Phylogenetic tree construction grouped the populations according to country of origin with the exception of the Medan population from Indonesia. The current gene flow was apparent within the regions but was restricted among the regions. The present study revealed that Indonesia and Thailand populations could be alternative centers of the gene pool, together with India. Consequently, regional action should be incorporated in genetic conservation and breeding efforts to develop new varieties with global acceptance.

## Introduction

Elephant foot yam (*Amorphophallus paeoniifolius* (Dennst.) Nicolson synonym *A*. *campanulatus* (Roxb.) Blume) is a perennial herbaceous diploid Araceae (2n = 2× = 26, 28) that is found across Australasian and African countries [1, 2] (S1 Fig). The plant is distributed from close to the coastal line to an altitude up to 900 m above sea level and adapts to low light intensities [3, 4]. The crop exhibits wide agroecological adaptation to dry and moist lands [1, 5] and is abundant under trees shading home gardens, mixed gardens, secondary forests and agroforestry, as well as open fields [4, 6, 7].

Mature underground corm and young shoots are used locally as important cuisine, medicine and disinfectants in many Asian countries [4, 7–15]. The corm can be industrially exploited for various enzymes and phytochemicals [13]. The starch has a low glycemic index [16–17] that may benefit diabetic individuals. According to Matthews [18], elephant foot yams might have been used as food in Southeast Asia since the prehistoric era. The starchy corm is harvested at the dormant stage in the dry or winter season [4], with productivity reaching 50–80 t ha^-1^ annually [7].

Along with vegetative growth in the rainy season, the plant releases some side-corms (cormels) [4]; thus, mature elephant foot yams are commonly surrounded by their smaller ramets. A single unisexual inflorescence emerges the fourth year after planting from the seed or cormel, and biannual flower bearing is common after the first flowering [4]. The reproductive system exhibits a dichogamous barrier to prevent selfing, and beetles assist with cross pollination in synchronous flowering [19–20], attracted by the rotten-meat odor released from the spadix [21]. Mature berries pertinently drop around mother plants; nevertheless, long-distance dispersal by birds has been reported [22].

*A*. *paeoniifolius* has two morphotypes—rough and smooth types—of petioles called *var*. *sylvestris* and *var*. *hortensis*, respectively [23], with numerous leaf morphological variations [24]. The roughness of the petiole becomes the main identifier for farmers because the rough type is associated with acrid corms; the smooth type also exhibits acridity at the immature stage [8, 24]. However, the morphotypes do not correspond to different genetic groups [25–27]. Therefore, breeding to enhance palatability to eliminate tuber acidity and reduce oxalic content becomes the main goal of local plant breeders.

The plant is assumed to be panmictic [1]. However, regional evaluation is lacking in that is unknown from the genetic background whether the current distribution reflects the plant’s natural range in Asia. The dispersal history of elephant foot yams has been poorly studied compared with that of other root crops such as taro [28] and sweet potato [29]. It has been speculated that *A*. *paeoniifolius* originated in India and was distributed by humans to other regions [30–31]. Local genetic assessment has been documented [25–27, 32–36]; nevertheless, information about the relationship among distant populations is still lacking. Long-distance dispersal by human participation has been speculated due to the plant being easily propagated clonally through cormels, corm skin or seeds and being handy as a travel logistic. However, the evidence for long-distance dispersal has not been evaluated. The objectives of this study were to elucidate the population structure and its relationship among populations from India, Indonesia and Thailand using microsatellite markers. Our understanding of the genetic structure of *A*. *paeoniifolius*, which exhibits a short life cycle and adapts to humid tropical areas in Asia, is essential for better conservation genetic and breeding for global palatability acceptance strategies.

## Materials and methods

### Ethics concern

Leaf samples were obtained using a method that was nondestructive to the mother plant. Sterile field tools were used to ensure that no disease transmission occurred among the plants during sampling.

All the dried leaf samples were collected by authorized persons in the country of origin—i.e., Kasetsart University and Royal Forest Department, Central Tuber Crops Research Institute Kerala, and Bogor Agricultural University and Indonesian Institute of Sciences for sampling in populations from Thailand, India and Indonesia, respectively. The collection procedures for each country were performed in compliance with the national regulation. In Thailand, the collection procedures followed “Act on Wildlife Reservation and Protection B.E. 2535 (1992)”, “Act on Plant Varieties Protection B.E. 2542 (1999)”, and “Act on Protection and Promotion of Traditional Thai Medicinal Intelligence B.E. 2542 (1999)”. In India, these procedures followed “Act on Wild Life (Protection) Amendment 39 (2006)”, and in Indonesia, these procedures followed “Act on Conservation of Living Resources and Their Ecosystems 5 (1990)” and “Act on United Nation Convention on Biological Diversity 5 (1994)”.

We declare that permissions to enter and collect samples from private lands, including farmer properties, were granted by the lands owner. The individuals who participated in this manuscript have agreed and provided written informed consent to publish these case details.

### Sampling collection

**Fig 1:**
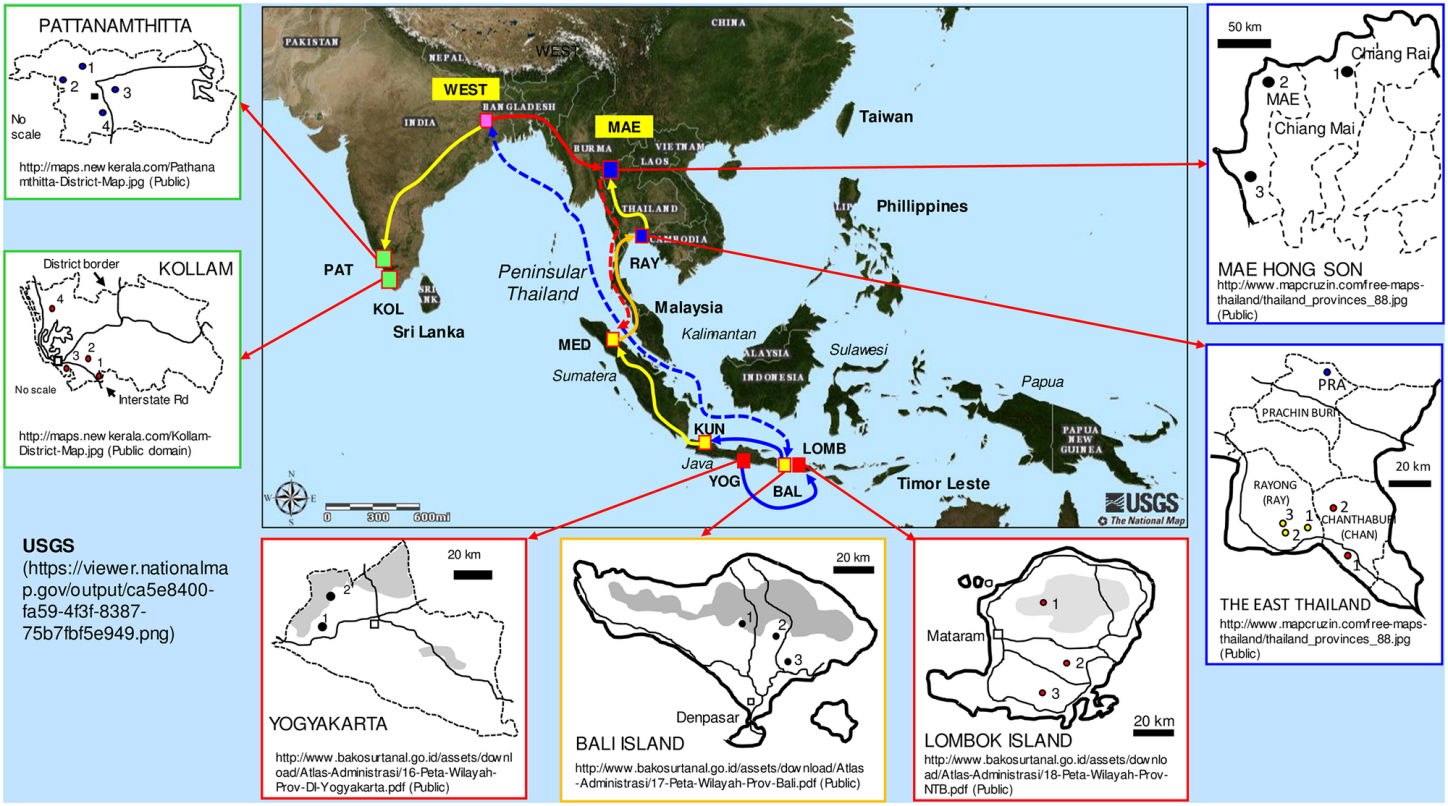
Study sites in India, Indonesia and Thailand. Filled dots denote study sites; provinces or islands are shown in insets; and numbers in an inset denote sampling sites. Bold lines are possible routes of gene flow. See S1 Table for the population codes and site descriptions.

During the rainy seasons (August to December) from 2003–2010, leaflets were collected from semi-wild and wild populations in India, Indonesia and Thailand (Fig 1). We considered particular countries in the present study as represented by the long history of elephant foot yam utilization. Efforts have been made to collect samples from other Asian countries including the Malaysian Peninsula and the Philippines but have been unsuccessful due to technical difficulties. In Indonesia, field visitation had also been conducted in southern Sumatera, Kalimantan, Sulawesi, Maluku and Papua islands according to a previous report [37]. However, in these Indonesian islands, the number of plant was very limited, and the lands owners of the particular sites clearly stated that the accessions were recently introduced from Java-Indonesia. Therefore, 29 populations were evaluated (S1 Table).

Elephant foot yam was identified based on a common description [24, 30]. It was collected randomly by considering the distance among plants larger than two meters, a petiole diameter larger than three centimeters, or a clear ancestor according to local authorities or land owners. Consequently, the coverage areas of a population varied depending on the genetic size from one-half to 100 hectares. In each population, 50 leaflets of most likely different genets (which are genetically identical individuals originating from asexual reproduction of a single mother plant) were collected, washed using mild detergent and then immediately stored in silica gel until DNA extraction.

The specimens of Indian samples are stored at the Central Tuber Crops Research Institute in Kerala, and Thai samples are maintained by Kasetsart University in Bangkok. Living specimens from the MED, KUN, YOGs and LOMBs populations are maintained at Bogor Agriculture University, Bogor-Indonesia, and the specimens from the BALs and LOMBs populations are maintained at Bali Botanical Garden, Bali-Indonesia.

### DNA extraction and microsatellite analysis

Molecular analysis including DNA extraction was carried out at the Laboratory of Forest Ecology, University of Tokyo, Japan, from 2010 to 2012, as listed step-by-step at http://dx.doi.org/10.17504/protocols.io.ht3b6qn. DNA was extracted using the modified CTAB method according to the protocol described by Lian et al. [38]. DNA from approximately one gram of dry leaflet was then preserved in a final volume of 200 μL of water and stored at -30°C until ready for use. Nineteen published microsatellites (SSR) [39] were evaluated, resulting in ten informative primers across populations, high polymorphism and unambiguous results.

PCR was carried out in a 5-μL tube, and a 0.5-μL aliquot of extract was used in each PCR amplification to approximately contain 5–20 ng of DNA. All SSR markers and DNAs were amplified with a PCR reagent mix of 0.5 μM forward primer, 0.1 μm reverse primer tailed with U-19 (Fastac Co, Tokyo-Japan), 0.5 μM U-19 primer labeled with Texas Red, 0.2 mM each dNTP, 1× PCR buffer (Mg^2+^ free, Applied Biosystems, Thermofisher Scientific, MA, USA), 2.5 mM MgCl_2_, and 0.5 U of AmpliTaq Gold DNA polymerase (Applied Biosystems). PCR (TAKARA PCR system, Japan) was performed using hot start as follows: one cycle of 9 min at 94°C, 30 s at the locus-specific annealing temperature (*T*_*a*_) and 1 min at 72°C; 38 cycles of 30 s at 94°C, 30 s at the locus-specific *T*_*a*_ and 1 min at 72°C; one cycle of 30 s at 94°C, 30 s at the locus-specific *T*_*a*_ and 5 min extension at 72°C; and ending at 4°C. We extended initial denaturation in PCR step to improve bands clarity.

Next, 1.5 μL of the PCR product was mixed with 3.5 μL of loading dye, denatured using the Thermal cycle TAKARA for five minutes at 95°C, and then placed on ice immediately for five minutes prior to loading. Two-microliter aliquots were then loaded onto a 6% denaturing polyacrylamide gel and run using the SQ-5500 sequencer (Hitachi Co., Tokyo). The band patterns were analyzed with FRAGYLS ver. 2 (Hitachi Electronics Engineering Co., Japan) and verified. Two bands were considered different alleles if the difference was two or larger than two base pairs considering all primers used were dinucleotides. In rare case, bands of different alleles were identified as homo alleles by FRAGYLS software due to the imperfect gel separation of a particular individual. In such cases, the bands needed adjustment in the FRAGYLS software. We adjusted the band size manually considering the band quality and identified the particular alleles as homo or hetero to avoid the bias estimate of FRAGYLS software.

### Statistical analysis

Multilocus genotypes were identified using CERVUS 3.0 [40–41]. The probability (P_SIB_<0.001) is the likelihood that the genotype of one individual will have the same ramet as that of a second individual; the ramet from one population was excluded. Thus, plants with the same SSR profile were considered one individual.

The number of alleles (*Na*) and the observed (*H*_*O*_) and expected heterozygosity (*H*_*E*_) were calculated. The allelic diversity, frequency of null alleles and Hardy-Weinberg equilibrium (HWE) tests were estimated using CERVUS 3.0 and GENALEX softwares [42]. GENEPOP software [43] was used to test the HWE for all loci and its linkage disequilibrium (LDE) using Fisher’s method. Pairwise *F*_*ST*_ (fixation index) and *F*_*IS*_ (inbreeding coefficient) for each allele and subpopulation *H*_*E*_ were estimated using GENALEX [42]. A pairwise genetic distance matrix using unbiased Nei's distance was also created [44]. *F*_*ST*_ was estimated using the program FSTAT 2.9.3 [45]. Population bottleneck was estimated based on 1000 replications using BOTTLENECK software [46]. A phylogenetic tree was constructed based on genetic distance matrices from allele frequencies using POPTREE with 1000 bootstrap replications [47].

AMOVA was conducted to examine genetic variation among regions, among populations, and among individuals. Genotypic differentiation was evaluated for each population by calculating the *P value* of the *F*_*ST*_ estimate. Values of *F*_*IS*_ and *F*_*ST*_ were estimated based on the formula of Weir and Cockerham [48]. The likely area of origin might be inferred from geographic distributions by counting the gene flow. The amount of gene flow (*Nm*) was estimated from the *F*_*ST*_ estimates using 9,999 permutations in GENALEX [42]. Genetic distance was defined as *F*_ST_/(1 − *F*_ST_) [49]. Isolation by distance was performed [50]. *F*_*ST*_ was classified as low (<0.05), moderate (0.05–0.15), high (0.15–0.25) and very high (>0.25) genetic differentiation [51]. The *F*_*ST*_ value ‘0’ indicates no population structuring or panmixia, while ‘1’ indicates the existence of perfect barriers of gene flow. Inbreeding was concluded in a particular population based on *F*_*IS*_ estimate [51].

Population structure was examined using STRUCTURE ver 2.3.4 [52], followed by the admixture model and option of independent allele frequency between populations. A burn-in length of 50,000 iterations was followed by 100,000 Markov Chain Monte Carlo (MCMC) iterations. The pre-evaluation population number (cluster), *K*, was set from 1 to 15, and the model was run for 10 independent simulations for each *K*. True *K* was selected according to the assignment of Evanno et al. [53].

## Results

### Genetic diversity

All loci produced highly polymorphic alleles across populations, and the polymorphic information content ranged from 0.620 (Ampa19) to 0.937 (Ampa06), with an average of 0.803. The *Na* ranged from 10 to 61 (average 26.7), and 267 alleles were generated from 10 loci (Table 1). All loci produced high heterozygosity (*H*_*O*_>0.600), except for Ampa10 and Ampa15. Locus Ampa15 had the lowest *H*_*O*_ (0.288) and *H*_*E*_ (0.351). Six of ten loci expressed excess heterozygosity. The average *F*_*IS*_, *F*_*IT*_ and *F*_*ST*_ for each locus were -0.032, 0.249, and 0.279, respectively. Most loci generated high to very high *F*_*ST*_ values (0.201 to 0.476), except for Ampa17 (*F*_*ST*_ = 0.191). All loci were in HWE (P<0.0000), demonstrating the suitability for population study. The frequency of null alleles from all loci ranged from 0.039 to 0.337. The loci Ampa10 and Ampa15 generated high null allele frequencies—i.e., 0.275 and 0.337, respectively. The LDE test for each locus pair across all populations was insignificant (P<0.0000)—except for the locus pairs Ampa04-Ampa07, Ampa07-Ampa15, Ampa07-Ampa19, Ampa15-Ampa16, and Ampa10-Ampa15, which exhibited low linkage equilibrium.

**Table 1:** Summary of 10 microsatellite loci across populations of elephant foot yam, *Amorphophallus paeoniifolius* (Dennst.) Nicolson.

Locus	Total allele	*H*_*O*_	*H*_*E*_	*F*_*IS*_ ^a^	*F*_*IT*_	*F*_*ST*_
**Ampa04**	26	0.610	0.610	0.001	0.321	0.320
**Ampa05**	33	0.838	0.691	-0.212	0.032	0.201
**Ampa06**	61	0.668	0.721	0.073	0.294	0.239
**Ampa07**	20	0.611	0.609	-0.003	0.262	0.265
**Ampa10**	33	0.524	0.575	0.089	0.372	0.311
**Ampa12**	18	0.673	0.583	-0.155	0.133	0.250
**Ampa15**	10	0.288	0.351	0.179	0.570	0.476
**Ampa16**	23	0.764	0.646	-0.183	0.092	0.232
**Ampa17**	26	0.650	0.633	-0.028	0.169	0.191
**Ampa19**	17	0.605	0.558	-0.084	0.250	0.308
**Mean**	26.7	-	-	-0.032	0.249	0.279

*H*_*O*_ observed heterozygosity, *H*_*E*_ expected heterozygosity, *F*_*IS*_ inbreeding coefficients, *F*_*ST*_ genetic differentiation of the total population, *F*_*IT*_ fixation index of individuals within the total population.

^a^ negative value indicates excess heterozygosity.

From 1,046 total accessions, 200 individuals had the same multilocus genotypes and were treated as ramet. Ramet existed in 11 populations—i.e., KUN, YOG1, YOG2, KOL1, KOL2, KOL3, KOL4, PAT1, PAT2, PAT3 and PAT4 (Table 2). All loci generated 146 private alleles (54.7% of total allele) that were distributed in 15 populations—i.e., BAL1, BAL3, CHA2, CTRI, KOL1, KUN, LOMB1, LOMB3, MAE1, MED, PAT1, PRA, RAY2, RAY3 and WEST. Indonesian populations contributed the largest number of private alleles (88 alleles), followed by Thai (33 alleles) and Indian (22 alleles) populations. The Indian and Thai populations shared 40% of the private alleles, the Indian and Indonesian populations shared 30% of the private alleles, and the Indonesian and Thai populations shared 20% of the private alleles. WEST with 11 alleles, PRA with 23 alleles and MED with 76 alleles from the Indian, Thai and Indonesian populations, respectively, were the main private allele contributors.

**Table 2:** Structure and population differentiation of *Amorphophallus paeoniifolius* (Dennst.) Nicolson revealed using 10 microsatellite loci.

Pop	*N*	*Ng*	*Na*	*N*_*E*_	*I*	*H*_*O*_	*H*_*E*_	*F*_*IS*_^a^
**1. RAY1**	40	38	10	3.58	1.543	0.609	0.705	0.134
**2. RAY2**	40	40	10	5.16	1.724	0.590	0.762	0.238
**3. RAY3**	40	40	7	3.55	1.389	0.488	0.683	0.276
**4. CHAN1**	40	40	9	4.47	1.566	0.447	0.717	0.375
**5. CHAN2**	39	39	10	4.72	1.682	0.490	0.725	0.319
**6. PRA**	40	40	8	4.08	1.514	0.468	0.694	0.335
**7. MAE1**	40	40	8	3.53	1.376	0.493	0.636	0.229
**8. MAE2**	40	40	7	2.92	1.264	0.454	0.625	0.262
**9. MAE3**	40	40	10	3.60	1.559	0.608	0.705	0.135
**10. KUN**	40	7	4	2.93	1.153	0.629	0.624	-0.037
**11. YOG1**	35	28	4	2.21	0.872	0.661	0.490	-0.252
**12. YOG2**	39	29	4	1.98	0.852	0.393	0.454	0.244
**13. LOMB1**	40	38	5	2.66	1.131	0.405	0.607	0.320
**14. LOMB2**	40	40	7	3.11	1.330	0.570	0.658	0.129
**15. LOMB3**	38	38	7	3.71	1.446	0.565	0.707	0.206
**16. BAL1**	35	30	7	2.83	1.198	0.502	0.598	0.213
**17. BAL2**	16	13	4	2.17	0.850	0.677	0.486	-0.268
**18. BAL3**	26	26	6	3.15	1.240	0.447	0.645	0.295
**19. MED**	30	30	11	5.73	1.710	0.435	0.683	0.366
**20. CTRI**	30	22	6	2.83	1.086	0.691	0.559	-0.267
**21. WEST**	40	39	7	3.58	1.329	0.599	0.643	0.037
**22. KOL1**	30	16	4	2.13	0.816	0.838	0.503	-0.617
**23. KOL2**	40	24	4	2.28	0.790	0.845	0.492	-0.759
**24. KOL3**	38	17	3	2.18	0.722	0.851	0.473	-0.839
**25. KOL4**	32	25	3	2.29	0.765	0.872	0.484	-0.852
**26. PAT1**	39	22	3	2.12	0.778	0.841	0.500	-0.669
**27. PAT2**	23	11	3	2.06	0.743	0.855	0.487	-0.712
**28. PAT3**	37	15	3	2.14	0.754	0.880	0.489	-0.834
**29. PAT4**	39	19	3	2.35	0.793	0.868	0.495	-0.812
**Total (mean)**	1046	846	(6)	(3.11)	(1.172)	(0.623)	(0.598)	(-0.084)

*N* sampling size, *Ng* number of genets, *Na* number of alleles, *N*_*E*_ number of effective alleles, *I* Shannon’s information index, *H*_*O*_ observed heterozygosity, *H*_*E*_ expected heterozygosity, *F*_*IS*_ subpopulation fixation index,

^a^ negative indicates excess heterozygosity.

The overall genetic diversity was high across regions as confirmed by morphological variation of the leaf and corm (S2 Table). The percentage values of molecular variance among regions, among populations, among individuals within population and among individuals across all populations were 16%, 11%, 8% and 65%, respectively. The Nei unbiased genetic distance ranged from 0.000 to 1.165, 0.124 to 1.474, and 0.000 to 0.606 within populations from Thailand, Indonesia and India, respectively (S3 Table). The genetic distance between Indonesian-Thai, Indonesian-Indian, and Indian-Thai populations was 0.694 to 1.964, 0.733 to 2.149, and 0.630 to 1.975, respectively.

### Population structure

The *Na* in each population ranged from 3 to 11 (average 6 allele), and the number of effective alleles ranged from 1.98 to 5.73 (average 3.11 allele) (Table 2). The diversity index (*I*) within a population ranged from low (0.722) to moderate (1.724). The average *H*_*O*_ and *H*_*E*_ were 0.623 and 0.596, respectively. Twelve of 29 populations expressed excess heterozygosity—i.e., three from Indonesia (KUN, YOG1, BAL2) and nine from India (CTRI, KOL1, KOL2, KOL3, KOL4, PAT1, PAT2, PAT3 and PAT4). Consequently, according to Allendorf [54], these twelve populations might severely bottleneck. Bottleneck estimate using the Wilcoxon test [46] showed nine populations—i.e., KUN, all KOLs and all PATs exhibited bottleneck. YOG1 and BAL2 populations expressed excess heterozygosity; however, it lacked bottleneck signatures. It is probable that YOG1 and BAL2 populations are affected by population expansion according to Putman et al. [55].

**Fig 2:**
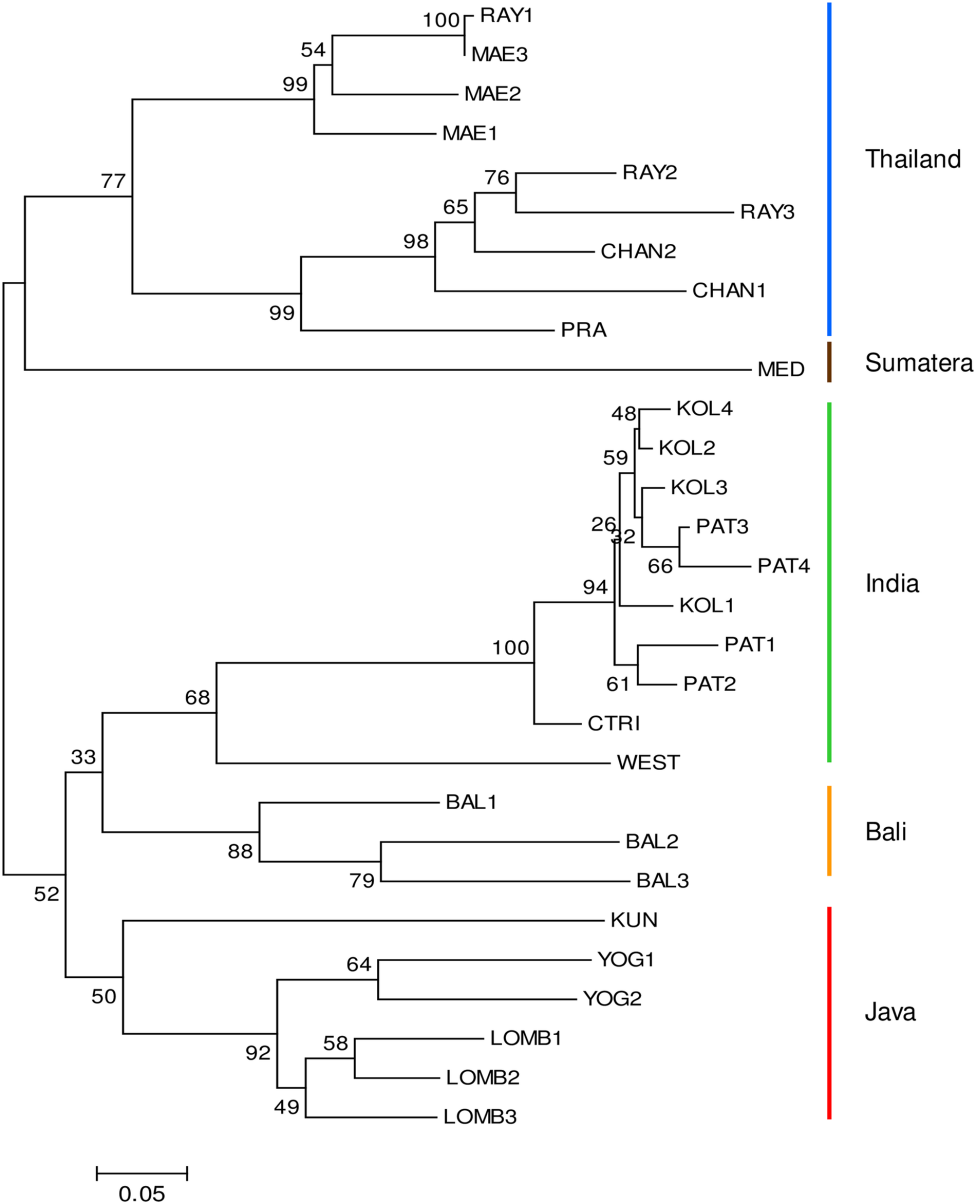
Dendrogram of the genetic distance among 29 *Amorphophallus paeoniifolius* populations using unrooted neighbor joining. The bar shows genetic distance.

The genetic differentiation (*F*_*ST*_) across regions was 0.274, which is classified as very high according to Hartl and Clark [51]. The *F*_*IS*_ value within a population was low (WEST and KUN, *F*_*IS*_ = 0.037) to very high (KOL3, F_IS_ = 0.839) (Table 2). The pairwise *F*_*ST*_ among populations ranged from low (*F*_*ST*_<0.000) to very high (*F*_*ST*_ >0.450) differentiation (S3 Table) but was significantly different from zero. The pairwise *F*_*ST*_ between Indian and Indonesian populations was larger than that between Indian and Thai populations—i.e., 0.178 to 0.431 and 0.182 to 0.337, respectively—while the *F*_*ST*_ between Indonesian and Thai populations was 0.153 to 0.391 (S3 Table). The *F*_*ST*_ values within Indian populations were low to high, and the WEST population was significantly structured relative to the others. Within Indonesian populations, *F*_*ST*_ was medium to very high, and high structuring existed in Bali (BAL1, BAL2 and BAL3) and Sumatera (MED) populations. The *F*_*ST*_ of Thai populations was low to high. Surprisingly, the RAY1 population in east Thailand had a similar population structure to that of the MAE3 population in north Thailand. Indian, Indonesian and Thai populations clustered together in 90% of the trees, and the Indian and Thai populations further clustered in 87% of the dendrogram (Fig 2). The India and Indonesian populations intimated their relationship within the larger cluster in 84% of the trees. In the rest of the tree, all Thai, BAL and LOMB populations maintained a relationship in 51% of the trees, and the MED clustered in 63% of the trees. Therefore, the MED seemed to be the population bridge between the Indonesian and Thai populations.

**Fig 3:**
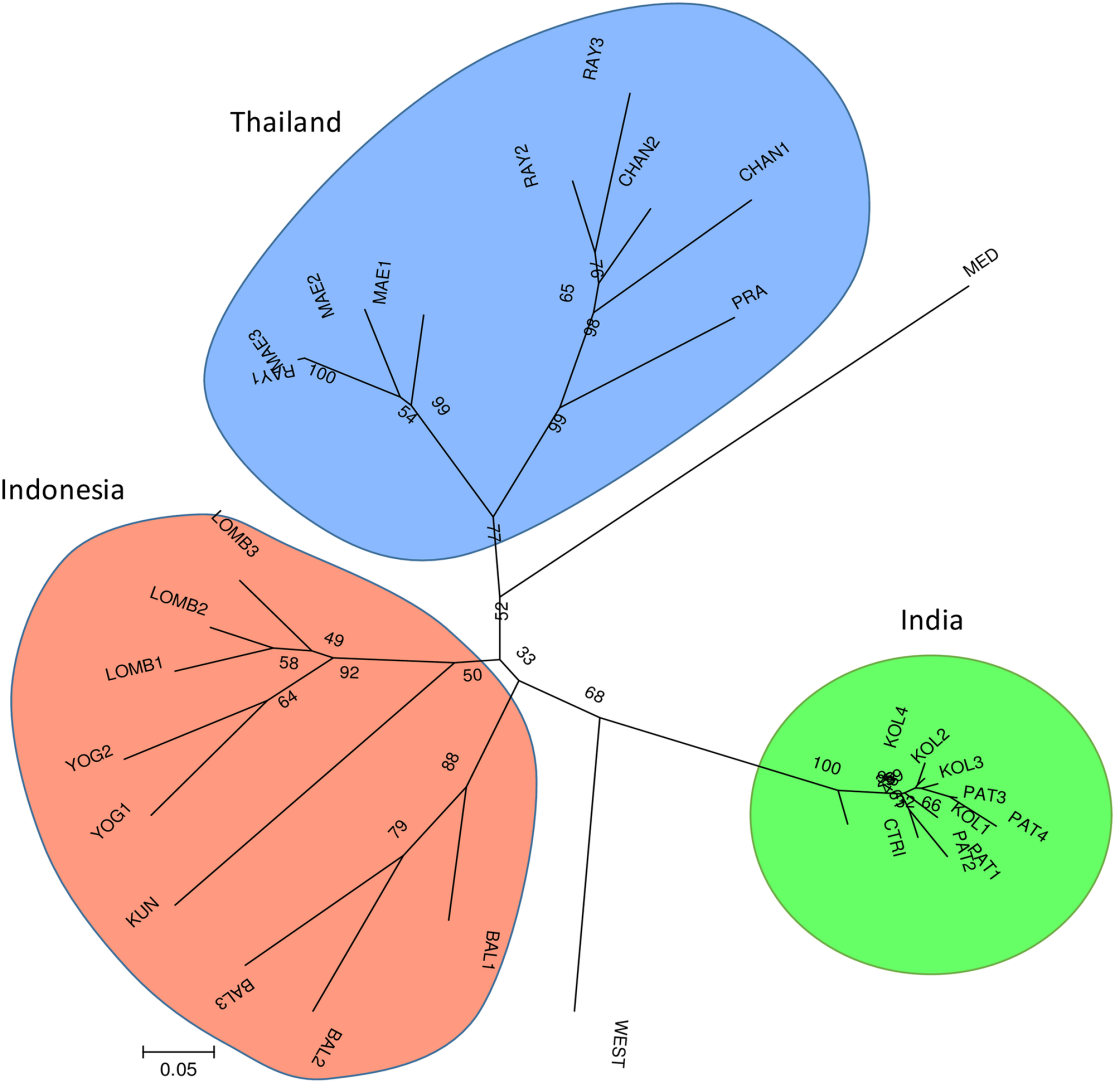
Phylogenetic tree among 29 *Amorphophallus paeoniifolius* populations from India, Indonesia and Thailand using 1,000 bootstrap replicates. The bar shows the genetic distance. The MED and WEST populations were considered out groups from the Indonesian and Indian populations, respectively.

Phylogenetic analysis grouped the populations into their respective regions (Fig 3); the bootstrap supported the relationship between Indonesia and Thailand based on clusters (100%), but it was weaker than between Indonesia and India (52%). The cluster concluded that BALs shared the same ancestor with all Indian populations and that the MED population shared the same ancestor with all Thai populations.

## Discussion

### Population dynamics and genetic conservation

**Fig 4:**
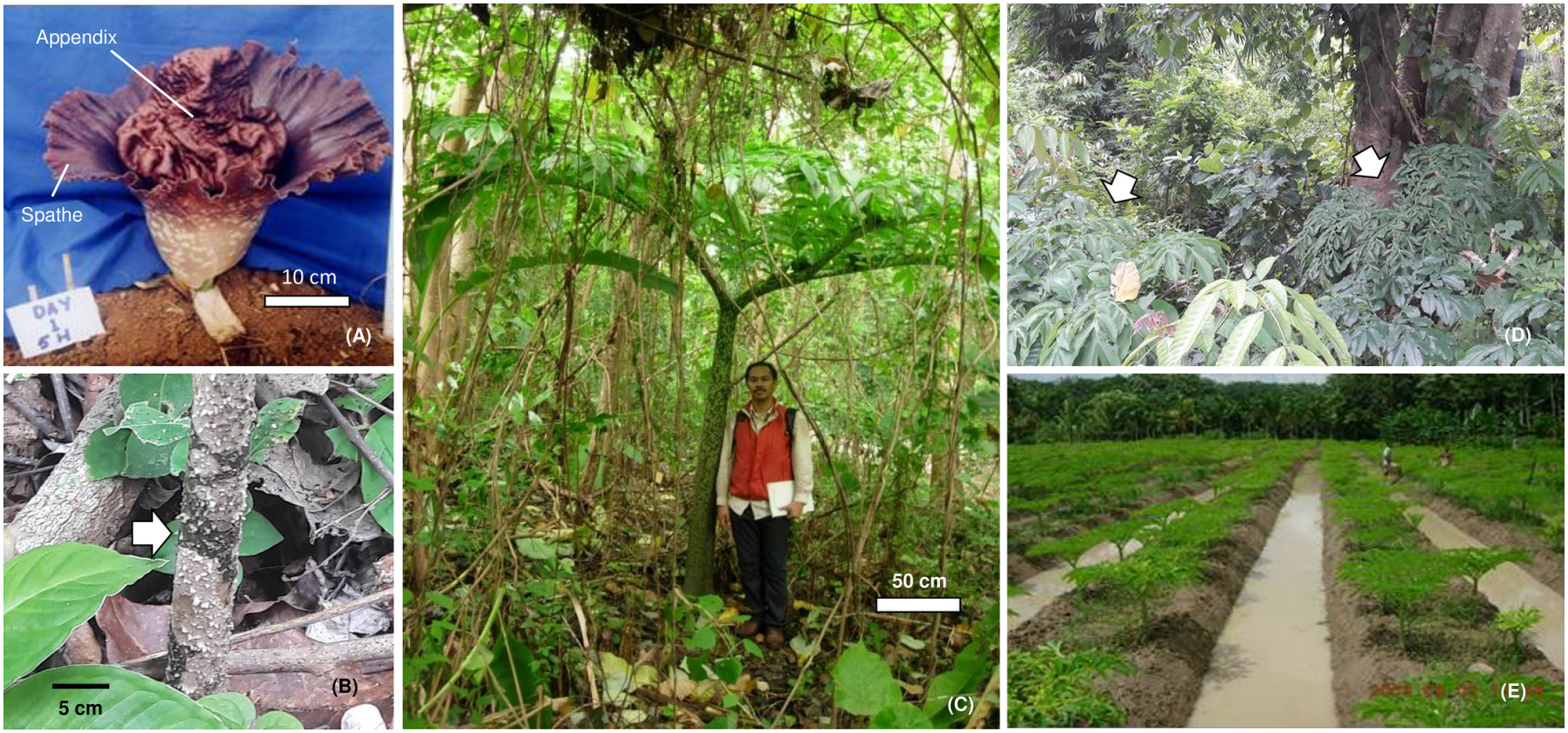
Inflorescence and cultivation of *Amorphophallus paeoniifolius*. (A) Inflorescence of elephant foot yams at anthesis. (B) Rough petiole of *A*. *paeoniifolius var*. *sylvestris*; the corm is considered less palatable in Lombok-Indonesia. (C) A large plant in Rayong Province, Thailand. (D) Semi-wild plants in an abandoned farm in Lombok-Indonesia. (E) Growing plants in an open lowland field in Kerala state, India.

The present study shows that genetic variation in elephant foot yams among different regions was evident (Table 2, S3 Table). The variation among regions could have arisen from degree of utilization and commercialization. The corms of elephant foot yams are commercially traded in the local markets of India but are less frequent in those of Indonesia and Thailand. Supported by high consumer preference and government attention, the India-supported Central Tuber Crops Research Institute (CTCRI) has become a leader in cultivation technology (Fig 4). High-yielding varieties with high palatability have been introduced by the Indian government, such as Sree Padma, Gajendra, Sree Athira (a hybrid), Bidhan Kusum and NDA-9 [7, 15]. Large commercial areas have been developed in India, including in Andhra Pradesh, Kerala, Maharashtra, and West Bengal [7, 31]. On the other hand, no commercial variety has been developed in Indonesia and Thailand, but substantial genetic conservation is available at Bogor Agriculture University (Indonesia), the Indonesian Center for Agriculture Biotechnology and Genetic Resources Research and Development (ICABIOGRAD- Indonesia), Bogor Botanical Garden (Indonesia), and Ministry of Agriculture and Cooperatives (Thailand) and Department of National Parks, Wildlife and Plant Conservation/Royal Forest Department (Thailand). Continuous utilization of high-yielding and high-palatability varieties could suggest that Indian populations, especially KOLs and PATs, have low allelic richness—consequently, population structuring occurs between Indian and Thai/Indonesian populations.

The utilization of elephant foot yams in Indonesian and Thailand has declined due to the increasing popularity of rice. In eastern Thailand, the plant is considered a weed. The decreased palatability of local peoples has led to reduced areas for elephant foot yam cultivation and conversely increased areas for semi-wild populations. In MAEs and RAYs of Thailand, some populations have been conserved due to underutilization, resulting in larger plant sizes (Fig 4C). A single elephant foot yam plant, as a perennial, enables the production of a 100-kg fresh corm [1]. Indian populations were dominated by plants one-meter in height with corms of approximately 3.0–3.5 kg. These facts demonstrate that a change in the local diet influences population features, especially plant size.

In a recent case in Indonesia, especially for LOMBs, intense intercropping programs in conservation forests using cash crops such as maize and upland rice threatened the habitat of the wild elephant foot yam population. The remaining populations were likely to have developed from the survivor after escaping from intense soil plowing. As a result, wild populations were mostly clumped along the forest edge, as well as on abandoned land, riverbanks or the edges of conservation forests (S1 Table).

Some populations constituted a larger number of rough petioles than smooth ones, probably because the excessive amount of plants with rough petioles in the population is a result of the consumer preference for plants with smooth petioles. The populations YOG1 and CHAN2, for example, consisted of approximately 65% plants with rough petioles. In KUN, although some farmers were enthusiastic to harvest the leaves of rough petioles for use as a disinfectant for fish ponds, the proportion of rough petioles was still high because leaf harvesting did not cause plant death. According to Shepherd et al. [56], bottlenecks are generated by the action of domestication, cultivation, introgression and extinction. It seems that people’s preference for a particular morphotype could cause drift in elephant foot yam populations. Azmi [57], however, noted that when seeds from an inflorescence were sown, some smooth, rough and intermediate forms of the petioles emerged. It is likely that selection and extent of cultivation for high palatability in YOG1, KOLs and PATs populations might increase *H*_*O*_ (Table 2) and reduce genetic diversity in the absence of cross breeding.

Within a single elephant foot yam population, it is common to observe *var*. *hortensis*, *var*. *sylvestris*, and *var*. *paeoniifolius* [58]. The general morphological properties of the studied population are presented in S2 Table. However, drift and reproductive success might affect the composition of a population. Clonal propagation is evident in a population as indicated by many ramets around a mother plant. A large corm enables the production of up to 43 cormels each year [4], which will develop into new ramets in subsequent growing seasons. On the other hand, seed propagation is restricted in some populations due to farmers cutting the flower spadix to avoid spreading the unpleasant rotting meat odor released at anthesis in Java and Bali cases and to prevent the seed set from experiencing hybridization or inbreeding. Unlike in Indonesia, farmers in Rayong (RAY) and its neighboring provinces in Thailand valued flower organs (Fig 4A) as local medicine and sold the seeds commercially. In the Amphoe Sai Yok market of Kanchanaburi Province, the seeds were sometimes available.

In Lombok Island and Mae Hong Son Province, many elephant foot yams seedlings grow around a tree base and in the cavity of dead branches of trees. It is probable that birds feed on the sweet pulp of mature berries and drop the seed, facilitating short-distance dispersal. Interestingly, during flooding events, some seeds disperse through river flow, similarly to dispersal in Rayong Province (RAY1). Spongy berries might enable the seeds to float and maintain their viability. The plant produces polyembryony seeds at a rate of up to 0.96%, and optimum germination is obtained at 6 weeks after harvest [57]. Although it is still preliminary, the long-distance dispersal of seeds through water bodies might be considered.

### Elephant foot yam dispersal among regions

The Mantel test showed no isolation by distance, indicating that the present distribution might reflect the gene flow. High genetic differentiation among regions but low genetic differentiation within regions indicated that gene flow was restricted among regions. The average migration rate (*m*) across regions was 1.126.

Microsatellite analysis concluded that geographic isolation between India and Thai populations is unlikely to be absolute, although the WEST population of India was separated from all northern Thailand populations (MAEs) by a large genetic distance (S3 Table). According to the local people, around the 18^th^ century, many Indians visited Mae Hong Son (MAE) to harvest teak timber that might include the elephant foot yams. Second, there is a high number of private alleles in the MED population that are shared with the Java (YOGs and KUN) and eastern Thai (RAYs) populations but a low number that is shared with Indian populations. The lack of influence of Indian alleles was demonstrated by the Ampa17 locus in Indonesian populations, indicating the uniqueness of the MED and LOMBs populations. The microsatellite profiles also indicated that the populations of KUN, YOGs, MED, LOMBs and RAYs share the same ancestor. Geographically, MED and RAYs were separated by the Malacca Strait, Malaysian Peninsula and Thai Peninsula (Fig 1). Maneenoon et al. [14] reported that elephant foot yams exist in the Thai Peninsula (Songkhla), while Phornvillay et al. [59] reported continuous variation in the morphological characteristics of *A*. *paeoniifolius* in Malaysia. It needs further clarification whether the peninsular populations have the intermediate genetic profile between the MED and RAYs populations.

**Fig 5:**
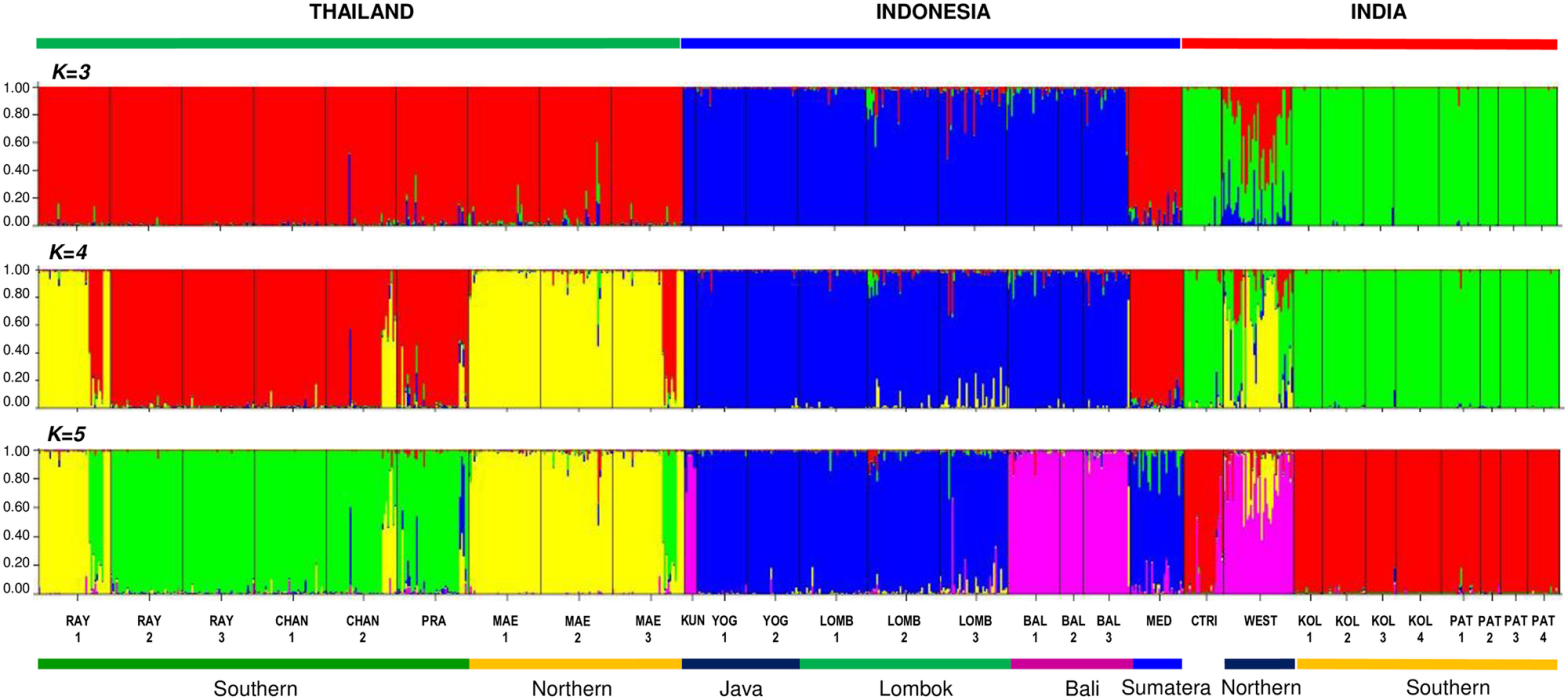
Model-based ancestry for each individual in *Amorphophallus paeoniifolius* populations with enforcement of the clusters K = 3, K = 4 and K = 5. The color codes represent the clusters of the STRUCTURE analysis.

The ancestry model did not support the panmixia population of elephant foot yams (Fig 5). The widely distributed population across many geographical barriers (S1 Fig) could indicate a high gene flow after cultural interrelationship among the regions.

The BALs clustered in one group with all Indian populations, and all Thailand populations clustered with the MED populations (Fig 5). Consequently, we propose that the populations of Indonesia with long-standing utilization could be an alternative center of diversity in the southern hemisphere. In this hypothesis, the gene flow was directed from Java to Bali and to India, contrary to the findings of Hetterscheid and Ittenbach [30]. From Bali, the gene flow was directed to Sumatera (MED) through KUN and finally to Thailand (Fig 1). It is generally accepted that the center of the crop origin exhibits larger genetic diversity. In the present study, Indian populations exhibited a lower genetic diversity than did Thai and Indonesian populations. Therefore, Indonesia and Thailand could be the gene pool instead of India.

Not surprisingly, Indonesian populations clustered with Indian populations, indicating the intimacy the cultures shared. Human migration from the Indian subcontinent with more agricultural vision to southern Asia has been studied [60–62]. It is probable that the interaction is most likely controlled because the sea route from India or Indonesia is mostly accessible during the dry season, the same time that the corms are available. Until recently, many Javanese and Balinese have maintained some cultural aspects similar to those of Indians, including a palate for elephant foot yams. Although it requires further clarification, the role of this plant in social relationships or cultural ceremonies could have been important during ancient times in Asia because many areas near sacred places such as the Borobudur temple in Java-Indonesia conserve elephant foot yam populations, similar to in India and Thailand.

### Elephant foot yam exchange within regions

There was a close genetic relationship among populations within regions, indicating intense exchange within regions. The mainland type of India and Thailand supported a high gene flow, contrary to that of archipelagic Indonesia. Southern Indian populations (KOLs and PATs) were likely descendants from northern territories (Fig 1). It has been speculated that the excess heterozygosity in Indian populations may be a result of out-crossing in the genus, although a small amount of inbreeding exists [27]. In Thailand, the genetic fraction most likely flowed from the southern to northern part of the country. The *m* within the Indian population was *Nm* = 4.369, but *Nm* = 1.789 and *Nm* = 0.654 within the Thailand and Indonesia populations, respectively. A tendency for elephant foot yam to be an underutilized crop in Indonesia and Thailand might be the present genetic barrier to its spread over the population within the particular region.

In Indonesia, LOMB populations could be a result of the founder effect of propagules moving out unidirectionally from YOGs (Fig 1)—as indicated by low pairwise *F*_*ST*_ values (*F*_*ST*_ = 0.152–0.220) (S3 Table). Similar gene flow possibly occurs from BALs to KUN populations and from KUN to MED populations. All site pairs of YOG and LOMBs, BALs and KUN, and KUN and MED are isolated by seas (Fig 1)—conversely, sea current pathways around those sites [63] do not correspond to the gene flow pathways. Thus, the gene flow among Indonesian populations most likely follows human migration.

In a simulation at a farm of Bogor Agriculture University, a founder population was established within four years supported by the effective propagation of cormlets and seeds. The successful transmigration program from Java and Bali to the outer islands from 1904 to 1980s [64–65] might have facilitated the recent distribution, although there is no particular report of the use of elephant foot yams in a formal food program. Subsequently, human migration from Java to Kalimantan, Sulawesi, Maluku and Papua islands along with crop propagates, including elephant foot yams, is well known. In the East Nusa Tenggara islands, the role of the Sabu people in the distribution of *Amorphophallus* has been elucidated [5]. Here, we speculate that the high mobility of Javanese, Bugis or Sabu people in the past could have affected the genetic structure of distal populations of elephant foot yams in Indonesia, including in the Philippines, northern Australia and Madagascar.

Bali populations (BALs) were important in Indonesia because they were linked tightly to the WEST population of India; similarly, the MED population was linked to the RAYs populations of Thailand. The LOMB populations, as neighbors of the BALs, genetically separated into a different sub group, but both populations shared a similar ancestor to KUN in west Java. Simultaneous reintroduction might exist in the Lombok, Bali and Rayong populations. Therefore, the effect of gene flow enables the maintenance and recovery of allelic richness [66].

Unexpectedly, the MED population had higher genetic differentiation than the other Indonesian populations—resulting in a different subgroup (Fig 3). We speculate that a fraction of the WEST population has flowed into MAEs and flowed further into MED (Fig 1). Subsequently, it cross breeds with the MED population originally from KUN—leading to high pairwise *F*_*ST*_ values (0.215–0.375) of MED to the other Indonesian populations (S3 Table).

Finally, the present study revealed large genetic diversity in the elephant foot yam population across and within regions. Regardless of the need to collect additional data from Peninsular Malaysia and Thailand, the present findings reveal the need to encourage regional action on conservation to broaden the genetic pools in breeding programs. The elephant foot yams exhibit adaptability to different water regions [67–68], different altitudes [1] and various cropping management practices [4]. The challenge in developing new varieties with wider palatability acceptance will secure future food security under global warming conditions, especially in tropical regions.

## Conclusions

Genetic diversity in *A*. *paeoniifolius* is considered high among Indian, Thai and Indonesian populations. The populations express excess heterozygosity and very high genetic differentiation. Most populations were grouped based on the country of origin. Indonesian populations were clustered into three sub groups, Thailand populations into one and India populations into one. It is likely that elephant foot yams are native to Southeast Asia and spread to other regions, after which their simultaneous domestication, natural cross breeding and local genetic improvement affected the genetic structuring of the current population. Indonesian and Thai populations could be alternative centers of diversity. Consequently, the genetic improvement and conservation of elephant foot yams in Asia should be based on collaboration among regions to obtain optimal benefits from greater genetic pools.

## Supporting information

S1 Fig Distribution map of *Amorphophallus paeoniifolius* in Africa, Asia and Australia. The native range of the species is indicated in red. The map is constructed according to the information from Jansen et al. [1], Hetterscheid and Claudel [2], Sugiyama et al. [37] and Yuzammi et al. [68].
(TIF)

S1 Table Sampling codes and site description of *Amorphophallus paeoniifolius* populations in India, Indonesia (IDN), and Thailand (THAI). (DOCX)

S2 Table Morphological characteristics of *Amorphophallus paeoniifolius* across populations. (DOCX)

S3 Table *F*_*ST*_ estimates and Nei unbiased genetic distance among 29 Asian populations. *F*_*ST*_ values for microsatellite loci are given below the diagonal line, and those for genetic distance are given above the diagonal line.
(XLSX)

S4 Table Allele size of 10 microsatellites from 29 Asian populations. (XLSX)
