# Supplementary figures and images for: Population structure of elephant foot yams (Amorphophallus paeoniifolius (Dennst.) Nicolson) in Asia
Source: PLoS One. 2017 Jun 28;12(6):e0180000. doi: 10.1371/journal.pone.0180000 (PMC5489206; doi:10.1371/journal.pone.0180000)

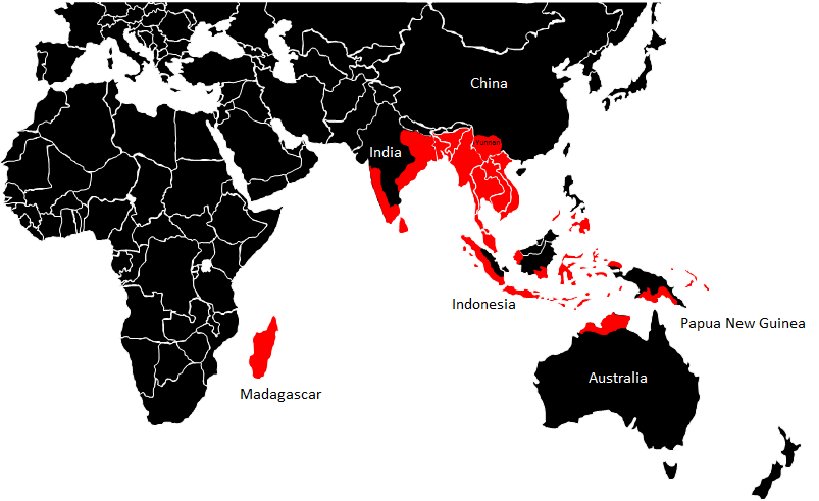

Supplement: S1 Fig — The native range of the species is indicated in red. The map is constructed according to the information from Jansen et al. [1], Hetterscheid and Claudel [2], Sugiyama et al. [37] and Yuzammi et al. [68]. (TIF) [file pone.0180000.s001.tif]
