# Supplementary material for: Population structure of elephant foot yams (Amorphophallus paeoniifolius (Dennst.) Nicolson) in Asia
Source: PLoS One. 2017 Jun 28;12(6):e0180000. doi: 10.1371/journal.pone.0180000 (PMC5489206; doi:10.1371/journal.pone.0180000)
Supplement: S1 Table — (DOCX) [file pone.0180000.s002.docx]

**Supplement Table 1. Sampling codes and site description of *Amorphophallus paeoniifolius* population in India, Indonesia (IDN), and Thailand (THAI)**

| **Pop** | **Coordinate** | **Village, State/Province, COUNTRY** | **Elevation (m)** | **Habitat** |  | **Pop** | **Coordinate** | **Village, State/Province, COUNTRY** | **Elevation (m)** | **Habitat** |
| --- | --- | --- | --- | --- | --- | --- | --- | --- | --- | --- |
| **KUN** | 6^o^ 59’ 31.45” S  108^o^ 29’ 05.60” E | Kuningan village, Kuningan, West Java, IDN | 490 | Escape, along Cisanggarung river |  | **PRA** | 14^o^ 21’ 54.72” N  101^o^ 50’ 28.76” E | Chambon Wan Kon Deng, Amphoe Nadi, Prachinburi, THAI | 500 | Taplaan National Park, abandoned farm |
| **YOG1** | 7^o^ 46’ 28” S  110^o^ 12’ 07” E | Ngentak, Kulonprogo, Yogyakarta, IDN | 150 | Escape along unintended farmland |  | **MAE1** | 19^o^ 24’ 22.25” N  98^o^ 55’ 12.19” E | Silana Nat Par, Chiang Dao-Mae Hong Son, THAI | 520 | Secondary conservation forest |
| **YOG2** | 7^o^ 43’07” S  110^o^ 19’26” E | Njelok, Kulonprogo, Yogyakarta, IDN | 190 | Escape along unintended farmland |  | **MAE2** | 19^o^ 23’ 11.58” N  97^o^ 57’ 53.53” E | Ban Mae Seket, Ampe Meang, Mae Hong Son, THAI | 600 | Conservation area of Royal Forest |
| **LOMB1** | 8^o^ 28’35” S  116^o^ 06’ 07” E | Pusuk Pemenang, Lombok Barat, West Nusatenggara, IDN | 300 | Conservation forest |  | **MAE3** | 19^o^ 19’ 42.47” N  97^o^ 57’ 57.33” E | Huay Sea Tao, Ampe Meang, Mae Hong Son, THAI | 600 | Wild orchid forest, abandoned farmland |
| **LOMB2** | 8^o^ 41’ 38” S  116^o^ 17’ 29” E | Kawu, Lombok Tengah, West Nusatenggara, IDN | 147 | Escape, bamboo forest |  | **CTRI^Z^** | - | All Indian collections, INDIA | - | Farmland |
| **LOMB3** | 8^o^ 32’04” S  116^o^ 19’ 47” E | Air Bukak, Lombok Tengah, West Nusatenggara, IDN | 549 | Conservation forest, Mt Rinjani |  | **WEST** | 20^o^ 15’ 49.61” N  85^o^ 50’ 05.40” E | Bhubaneswar, West Bengal, INDIA | 50 | Escape population |
| **BAL1** | 8^o^ 20’ 27.60” S  115^o^ 11’ 22.13” E | Desa Perian, Baturiti-Tabanan, Bali, IDN | 780 | Escape, along Tabanan river |  | **KOL1** | 8^o^ 49’ 13.28” N  76^o^ 45’ 15.39” E | Rovandu village, Kollam dist, Kerala, INDIA | 65 | Farmland, abandoned |
| **BAL2** | 8^o^ 22’ 59.30” S  115^o^ 20’ 08.99” E | Desa Pakraman Basanggabu, Tampak Siring- Gianyar, Bali, IDN | 730 | Escape, grass land near home garden |  | **KOL2** | 8^o^ 49’ 18.10” N  76^o^ 45’ 06.40” E | Karunaga pally, Kollam dist, Kerala, INDIA | 60 | Farmland, abandoned |
| **BAL3** | 8^o^ 36’ 11.03” S  115^o^ 16’ 55.05” E | Desa Batubulan, Kec Sukowati, Gianyar, Bali, IDN | 50 | Escape, Funeral place |  | **KOL3** | 8^o^ 49’ 06.78” N  76^o^ 44’ 59.17” E | Karunaga pally, Kollam dist, Kerala, INDIA | 50 | Farmland edge, abandoned |
| **MED** | 3^o^ 14’ 45.48” S  98^o^ 32’ 11.16” E | Sibolangit, Deli Serdang-Medan, North Sumatra, IDN | 800 | Sibolangit conservation forest |  | **KOL4** | 8^o^ 48’ 33.73” N  76^o^ 44’ 14.95” E | Karunaga pally, Kollam dist, Kerala, INDIA | 55 | Lowland farm, abandoned |
| **RAY1** | 12^o^ 50’ 45.38” N  101^o^ 39’ 20.19” E | Utong, Ampe Klang; Banna district, Ra Yong, THAI | 35 | River bank, flooded sometimes |  | **PAT1** | 9^o^ 33’ 51.74” N  76^o^ 44’ 07.90” E | Chadaya magalam, Pattanamthitta, Kerala, INDIA | 70 | Farmland, abandoned |
| **RAY2** | 12^o^ 44’ 34.24” N  101^o^ 25’ 36.30” E | Utong, Banna, Amphoe Klaeng, Ra Yong, THAI | 350 | Longkong orchard |  | **PAT2** | 9^o^ 33’ 49.48” N  76^o^ 44’ 04.17” E | Pampady village, Pattanamthitta, Kerala, INDIA | 70 | Farmland, abandoned |
| **RAY3** | 12^o^ 44’ 31.35” N  101^o^ 25’ 35.14” E | Kao Tah Chud, Mueng, Sam Nak Tong Amphoe, Ra Yong, THAI | 400 | Secondary forest, Mt. Yaida |  | **PAT3** | 9^o^ 33’ 47.22” N  76^o^ 43’ 56.90” E | Thumpamon, Pattanamthitta, Kerala, INDIA | 75 | Lowland, abandoned |
| **CHAN1** | 12^o^ 36’ 26.85” N  102^o^ 00’ 26.05” E | Nernsoang, Kao Wua, Tha Mai Amphoe, Chantaburi, THAI | 150 | Farm, mix home garden |  | **PAT4** | 9^o^ 34’ 07.09” N  76^o^ 43’ 28.89” E | Omallur village, Pattanamthitta, Kerala, INDIA | 75 | Farmland, abandoned |
| **CHAN2** | 12^o^ 59’ 03.09” N  101^o^ 54’ 27.32” E | Kao Cha Mao Kaung, Kaen Hang Maew, Sam Phi Nong Amphoe, Chantaburi, THAI | 200 | Mangosteen and Rambutan Orchard |  |  |  |  |  |  |
