# Supplementary material for: Population structure of elephant foot yams (Amorphophallus paeoniifolius (Dennst.) Nicolson) in Asia
Source: PLoS One. 2017 Jun 28;12(6):e0180000. doi: 10.1371/journal.pone.0180000 (PMC5489206; doi:10.1371/journal.pone.0180000)
Supplement: S2 Table — (DOCX) [file pone.0180000.s003.docx]

**Supplement Table 2. General morphological characteristics of *Amorphophallus paeoniifolius* in India, Indonesia and Thailand populations**.

| **Main characters** | **Description** | **Distribution in population^a^** |
| --- | --- | --- |
| **Petiole** |  |  |
| - Color | - Light green | India, Indonesia, Thailand |
|  | - Green | India, Indonesia, Thailand |
|  | - Green-pinkish | KUN, YOG2 |
|  | - Green-greyish | YOG1, YOG2 |
|  | - Dark green | India, Indonesia, Thailand |
|  | - Blackish green | Indonesia, Thailand |
|  | - Blackish pinkish | KUN, BALs, LOMBs |
| - Maximum height (cm) | - < 150 cm | India, Indonesia, Thailand |
|  | - > 200 cm | RAYs, MAEs, KUN, MED |
| - Roughness | - Smooth | India, Indonesia, Thailand |
|  | - Rough | India, Indonesia, Thailand |
|  | - Very rough | LOMBs, MED |
| - Spot size on petiole | - Small (diameter <2 cm) | India, Indonesia, Thailand |
|  | - Large (diameter ≥ 2 cm) | India, Indonesia, Thailand |
| - Additional color at tripartite branch | - Absent | India, Indonesia, Thailand |
|  | - Present (pink, black) | KUN, YOGs, BAL1, MED |
| - Sap color | - Colorless | India, Indonesia, Thailand |
|  | - Pinkish | KUN, YOGs, BAL1, MED, RAYs |
| **Leaf blade** |  |  |
| - Leaflets size | - Small (width < 5 cm) | India, Indonesia, Thailand |
|  | - Large (width ≥ 5 cm) | India, Indonesia, Thailand |
| - Shiny leaflets | - Absent | India, Indonesia, Thailand |
|  | - Present | India, Indonesia |
| **Corm**^b^ |  |  |
| - Cormels production | - Absent | India, Indonesia, Thailand |
|  | - Abundant | Indonesia, Thailand |
| - Corm shape | - Globose | India, Indonesia |
|  | - Depressed globose | India, Indonesia, Thailand |
| - Flesh color | - White | India, Indonesia, Thailand |
|  | - Light yellow | India, Indonesia, Thailand |
|  | - Light pink | India, Indonesia, Thailand |
|  | - Pink | KUN, MED, MAEs |
| - Corm acridity^c^ | - Non acrid | India, Indonesia, Thailand |
|  | - Acrid | India, Indonesia, Thailand |
|  | - Very acrid | BALs, LOMBs, MED |

^a^ Particular characters belong to general population, otherwise mentioned, ^b^ obtained from 3-5 corms at late stage before dormant, ^c^ checked qualitatively by back-hand touching: very acrid remains itchy for more than 1 hours.
